# The free neurovascular transverse wrist crease flap for repairing soft tissue defects of the fingers: clinical outcomes of multiple centers

**DOI:** 10.1186/s13018-019-1444-y

**Published:** 2019-11-14

**Authors:** Zhi-Qiang Fan, Bao-Fu Yu, Qi Zeng, Bo Cai, Guo-Ming Xia, Sheng-Hui Huang

**Affiliations:** 1 Department of Orthopaedic Surgery, Jiangxi Provincial People’s Hospital, Nanchang University, 152 Ai Guo Road, Nanchang, 330006 Jiangxi People’s Republic of China; 2 Department of Hand and Upper Extremity Surgery, Jingan Branch of Huashan Hospital, Fudan University, Shanghai, China; 3 Department of Plastic Surgery, Jiangxi Provincial People’s Hospital, Nanchang University, Nanchang, Jiangxi China; 4 Department of Orthopaedic Surgery, Nanchang Shuguang Hand and Foot Surgery Hospital, Nanchang, Jiangxi China; 5 Department of Orthopaedic Surgery, The First Affiliated Hospital of Nanchang University, Nanchang, Jiangxi China; 6 Department of Orthopaedic Surgery, The Second Affiliated Hospital of Sun Yat-sen University, Guangzhou, Guangdong China; 7 Department of Orthopaedic Surgery, The Seventh Affiliated Hospital of Sun Yat-sen University, Guangzhou, Guangdong China

**Keywords:** Transverse wrist crease flap, Soft tissue defects of the fingers, Neurovascular reconstruction, Multiple centers

## Abstract

**Background:**

Flap transplantation is often needed for soft tissue defects of the fingers that cannot be closed directly. Sensory reconstruction of the fingers is important for patients to recover feelings. In this study, we report clinical outcomes of using free neurovascular transverse wrist crease flap for repairing finger defects from multiple centers.

**Methods:**

This case series includes 72 consecutive patients with finger defects between June 2013 and June 2016 from multiple centers. A neurovascular transverse free radial artery superficial palmar branch flap, named transverse wrist crease flap, was designed to reconstruct soft tissue defects of the fingers with microvascular anastomosis. When there were soft tissue defects of the fingers with digital nerve defect, end-to-end neurorrhaphy between the median palmar cutaneous branch and the digital nerves was also performed. The donor incision was sutured directly. All the patients were followed-up for more than 24 months.

**Results:**

The soft tissue defects of the fingers were all completely covered with this free neurovascular transverse wrist crease flap, and the flaps in 71 patients survived completely without ischemia. Vascular crisis appeared in one case, and the wound healed gradually after changing wound dressing for nearly 1 month. Slight infections of wounds appeared in eight cases. There were no complications in the donor site, like infection and poor wound healing. At the last follow-up, the mean static two-point discrimination was 9.6 ± 2.4 mm on the injured finger and 4.5 ± 0.8 on the contralateral corresponding finger. The motion range of the distal interphalangeal joint and proximal interphalangeal joint on the injured finger were 72.5 ± 23.3% and 78.7 ± 32.5% of the contralateral corresponding finger, respectively. Patient self-evaluations were good in 53 cases and fair in 19 cases.

**Conclusions:**

The results indicate that the free neurovascular transverse wrist crease flap is a good choice for repairing soft tissue defects of the fingers.

**Level of evidence:**

Therapeutic IV

## Background

Soft tissue defects of the fingers with bone, tendon, or joint exposure are common injuries in hand traumas [1, 2]. To preserve finger length and function, flap transplantation rather than skin graft is usually performed to reconstruct the defective soft tissue. Currently, there are several kinds of flaps available for covering defects of the fingers, like V–Y advancement flaps, rotation flaps, and cross-finger flaps [3, 4]. However, each of these techniques has its limitations and cannot be applied to all kinds of finger defects. Besides, some of these flaps can be only used to repair very small defects, and some require additional skin graft procedure. In 2003, a transverse free radial artery superficial palmar branch (RASPB) flap, named transverse wrist crease flap, was firstly reported to be applied to reconstruct the finger defects [5]. Subsequently, there are several other studies reporting the clinical effects of this flap for reconstructing finger defects. But these studies only include less than 15 cases and reported limited experience of just single hospital. The safety and clinical effects are still needed to be validated with further studies including more patients and centers.

In this study, we report the clinical outcomes of the free neurovascular transverse wrist crease flap for reconstructing soft tissue defects of the fingers in 72 consecutive patients from multiple centers. We hope that this study will enable hand surgeons to increase the recognition of this technique.

## Methods

### Patients

A retrospective review was conducted on patients who had soft tissue defects of the fingers and received the transverse wrist crease flap for reconstruction between June 2013 and June 2016 at Jiangxi Provincial People’s Hospital, Jingan Branch of Huashan Hospital attached to Fudan University, Nanchang Shuguang hand and foot surgery Hospital, The First Affiliated Hospital of Nanchang University, The Second Affiliated Hospital of Sun Yat-sen University, and The Seventh Affiliated Hospital of Sun Yat-sen University, China. There were 72 emergent patients who were included in this study, including 53 males and 19 females. The average age was 42.5 ± 14.4 years (range, 19–62 years). Among all the patients, 31 cases underwent avulsion injuries, 30 cases underwent crush injuries, and 11 cases underwent twisting injuries. Seventy-one patients had only one finger undergoing this flap procedure, and the defective fingers included 21 thumbs, 15 index fingers, 24 middle fingers, 6 ring fingers, and 5 little fingers. One special patient had 3 fingers (middle finger, ring finger, and little finger) severely injured and underwent 3 flap procedures (one RASPB flap and two artery perforating branch flaps from forearm). Demographic characteristics of the patients are shown in Table 1.

**Table 1:** Demographic characteristics of the patients

Characteristic	
Sex, no. (%)	
Male	53 (73.6)
Female	19 (26.4)
Age, years	42.5 ± 14.4
Injury type, no (%)	
Avulsion injury	31 (43.0%)
Crush injury	30 (41.7%)
Twisting injury	11 (15.3%)
Injured finger treated with RASPB flap, no (%)	
Thumb	21 (29.2%)
Index finger	15 (20.8%)
Middle finger	25 (34.7%)
Ring finger	6 (8.3%)
Little finger	5 (6.9%)
Soft tissue defect (length × width: cm × cm)	
Mean, 2.3 ± 1.5 × 3.8 ± 1.7	Range, 1.8–2.9 × 2.6–4.5
Time from injury to surgery, hours	
Mean, 10.4 ± 4.5	Range, 6–15
Flap area (length × width: cm × cm)	
Mean, 2.6 ± 1.2 × 3.6 ± 1.5	Range, 2.0–3.1 × 2.9–5.0
Combined injury, no.	
Fractures	15
Tendon rupture	11
Digital nerve injury	4
Joint dislocation	3
Operation time, minutes	
Mean, 126 ± 42	Range. 105–150

### Surgical technique

The RASPB usually divides from the radial artery at 1.0–2.0 cm proximal to the distal wrist crease [6], with mean diameter at its original site being 1.2 mm (range, 1.0–1.4 mm), which is similar to the diameter of the digital artery [7]. The RASPB then descends along the ulnar side of scaphoid tubercle [8] and commonly goes together with one or two venae comitantes [9]. At the forearm, one or two subcutaneous superficial veins with appropriate size and length were chosen as the returning veins for the RASPB flap. The sensory innervation of this flap is the palmar cutaneous branch of the median nerve, which was also included in this flap for innervation [10, 11]. Doppler ultrasound was used to detect the path of the RASPB before the surgery. The Doppler ultrasound was performed with an EPIQ 7 duplex device (Philips, Best, Netherlands). According to the Doppler ultrasound blood flow signal, we marked the RASPB path from its origin with a water-resistant pencil.

**Fig. 1:**
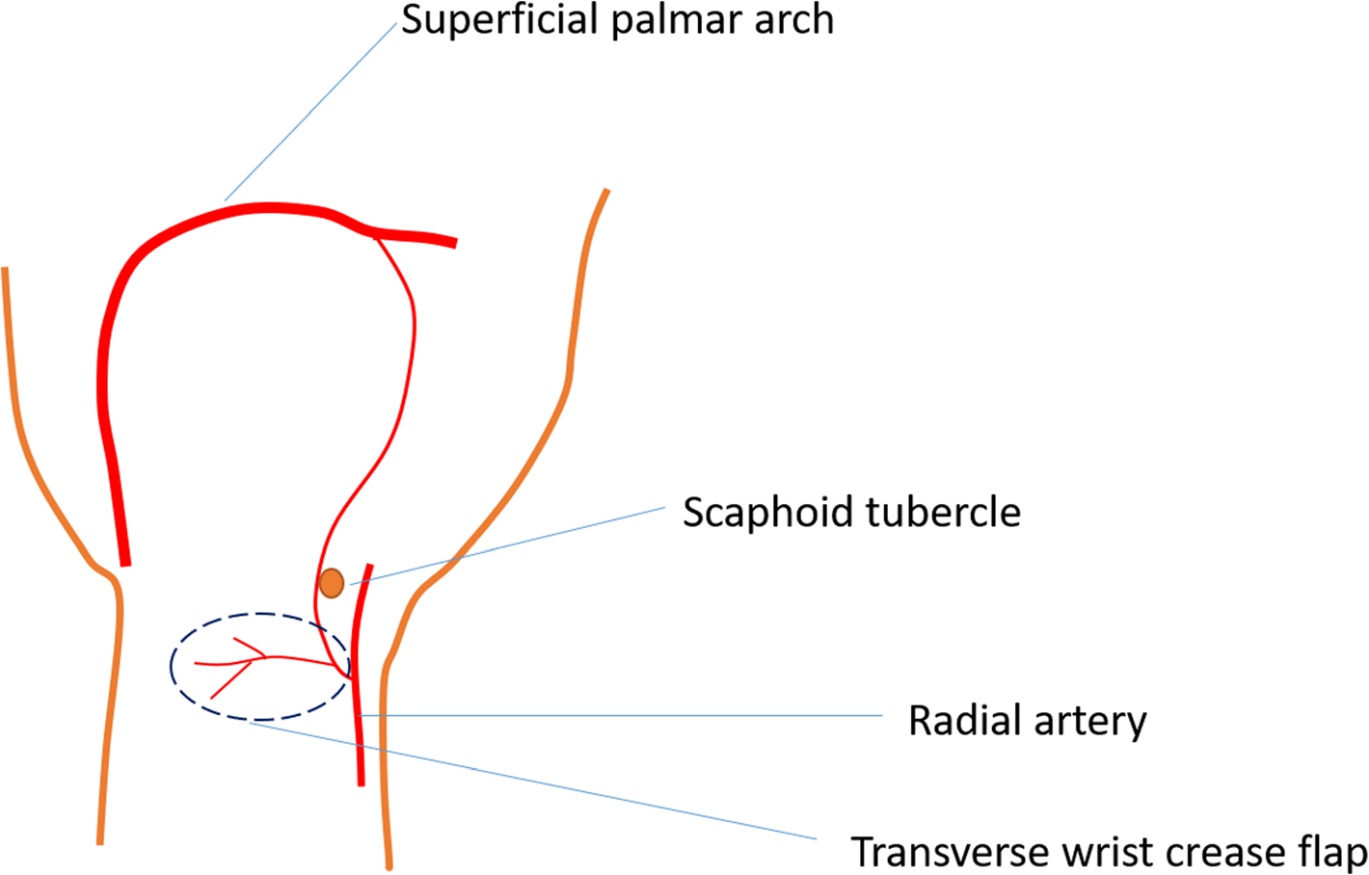
Illustration of the transverse wrist crease flap

**Fig. 2:**
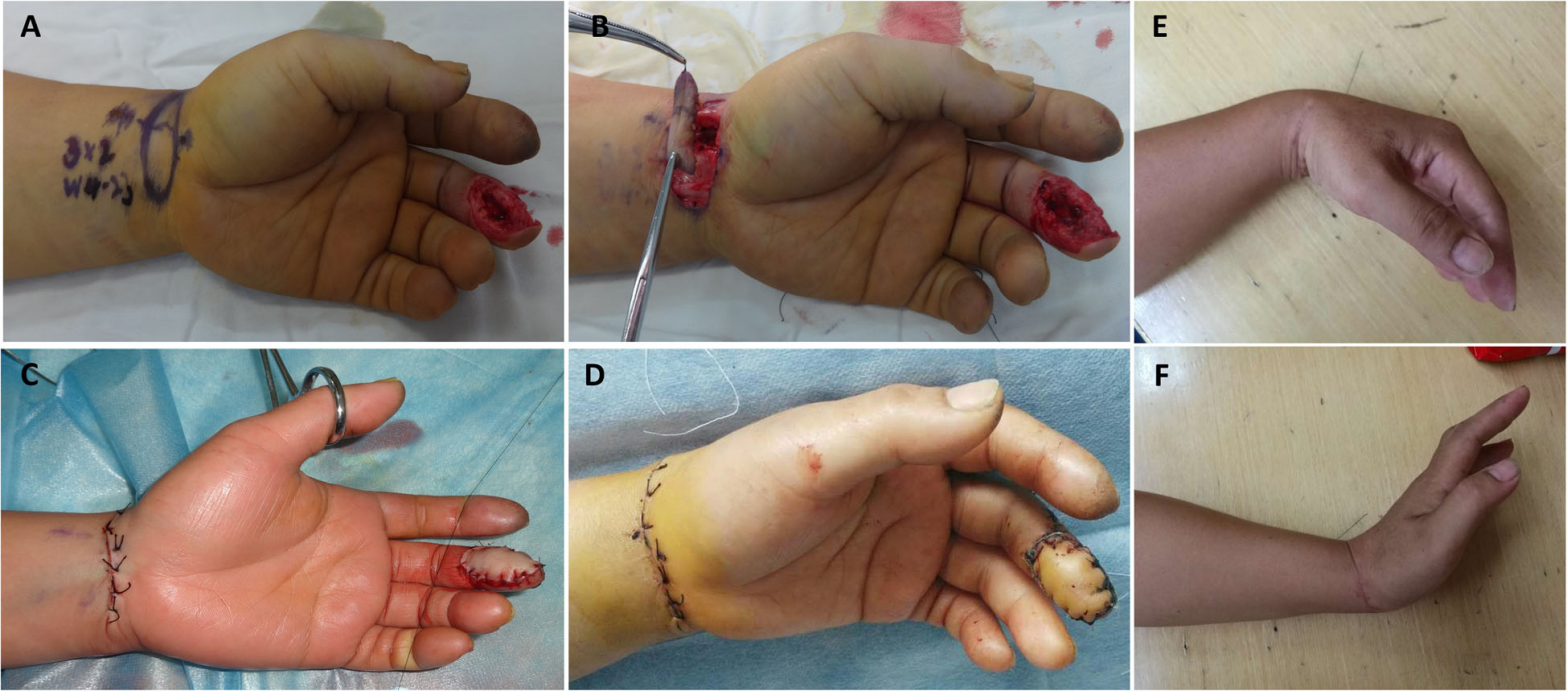
One case underwent the transverse wrist crease flap. **a** The middle finger had soft tissue defect with the tendon and bone exposed. A transverse wrist crease flap was designed according to the wound. **b** The transverse wrist crease flap was harvested. **c** The wound was covered with the flap, and the donor site was closed primarily. **d** After postoperative 14 days, the flap survived, and the wound healed properly. **e**, **f** After postoperative 24 months, the function of the donor site was not obviously affected

The procedure was performed under brachial plexus block anesthesia, and an air tourniquet is used to compress the upper arm of the injured side to achieve a bloodless surgical field with an approximate pressure of 260 mmHg. The injured fingers were debrided firstly, and the area of soft tissue defects was measured with a ruler. The digital artery and digital veins were dissected and marked for receptor vessels. The fractures were treated by closed reduction and fixed with K-wires. The rupture tendon was evaluated and repaired routinely before the flap procedure. According to the measured size and shape of the soft tissue defects, the free neurovascular transverse wrist crease flap was designed with 110% size of the defective area. The medium wrist crease was used as the axis of the flap [12–14] (Fig. 1). The RASPB origin is dissected by incising the radial margin of the flap firstly. Then, the proximal margin of the flap was incised, and superficial volar veins with sufficient length were dissected. The subfascial level was dissected subsequently. Under the deep fascia, the RASPB was dissected from the ulnar side to the radial side and distally [9]. There are usually more than two cutaneous perforators of the RASPB in the flap. The flap included RASPB, subcutaneous superficial veins, and the median nerve palmar cutaneous branch. Then, the donor site incision was sutured primarily (Fig. 2).

To check the blood supply of the flap, the tourniquet was temporarily released. When ischemic flaps appear, we could check if there was a twist in the pedicle of the flap. When the circulation was confirmed, the flap pedicle was cut and the free flap was located at the defective site. End-to-end anastomosis was performed from RASPB to the proper digital artery and the subcutaneous superficial vein to the digital dorsal vein with 11-0 Prolene sutures with a microscope. End-to-end neurorrhaphy was also performed from the median palmar cutaneous branch to the digital nerves with a microscope, when it was necessary, like repairing the end defect of finger. Generally, when facing soft tissue defects of the fingers with digital nerve defect, we would perform end-to-end neurorrhaphy of the median palmar cutaneous branch and the digital nerves. The wound was closed with 5-0 Prolene sutures. The wrist and injured fingers were immobilized for 2 weeks with a short arm plaster cast. After the surgery, baking lamp irradiation, low molecular heparin, narceine, and dextran were commonly used for 1 week in our hospital. Rehabilitations function exercises were routinely prescribed at postoperative 2–3 weeks according to injure conditions like fractures or tendon rupture.

### Outcome evaluation

All patients were followed up regularly (every 1–3 months). The range of motion (ROM) of the distal interphalangeal joint and proximal interphalangeal joint on the injured fingers were evaluated at the final follow-up. When the injured finger was the thumb, the metacarpophalangeal joint and the interphalangeal joint were evaluated instead. Static two-point discrimination was used to assess the sensation in the flap with Semmes-Weinstein nylon monofilament. Referring to the modified American Society of Surgery of the Hand Guidelines, it is considered to be excellent (≤ 6 mm), good (6–10 mm), fair (11–15 mm), and poor (≥ 15 mm) [15], respectively. A visual analog scale ranging from 0 to 10 was based on patients’ return to their previous work, the appearance of the donor and recipient sites, and functional recovery as referred to previous study [16].The patients were instructed to self-evaluate their conditions with this visual analog scale. The results were divided into excellent (10 scores), good (8–9 scores), fair (5–7 scores), poor (1–4 scores), or very poor (0 scores) [8], respectively. The contralateral corresponding finger was evaluated at the same time as well.

## Results

**Fig. 3:**
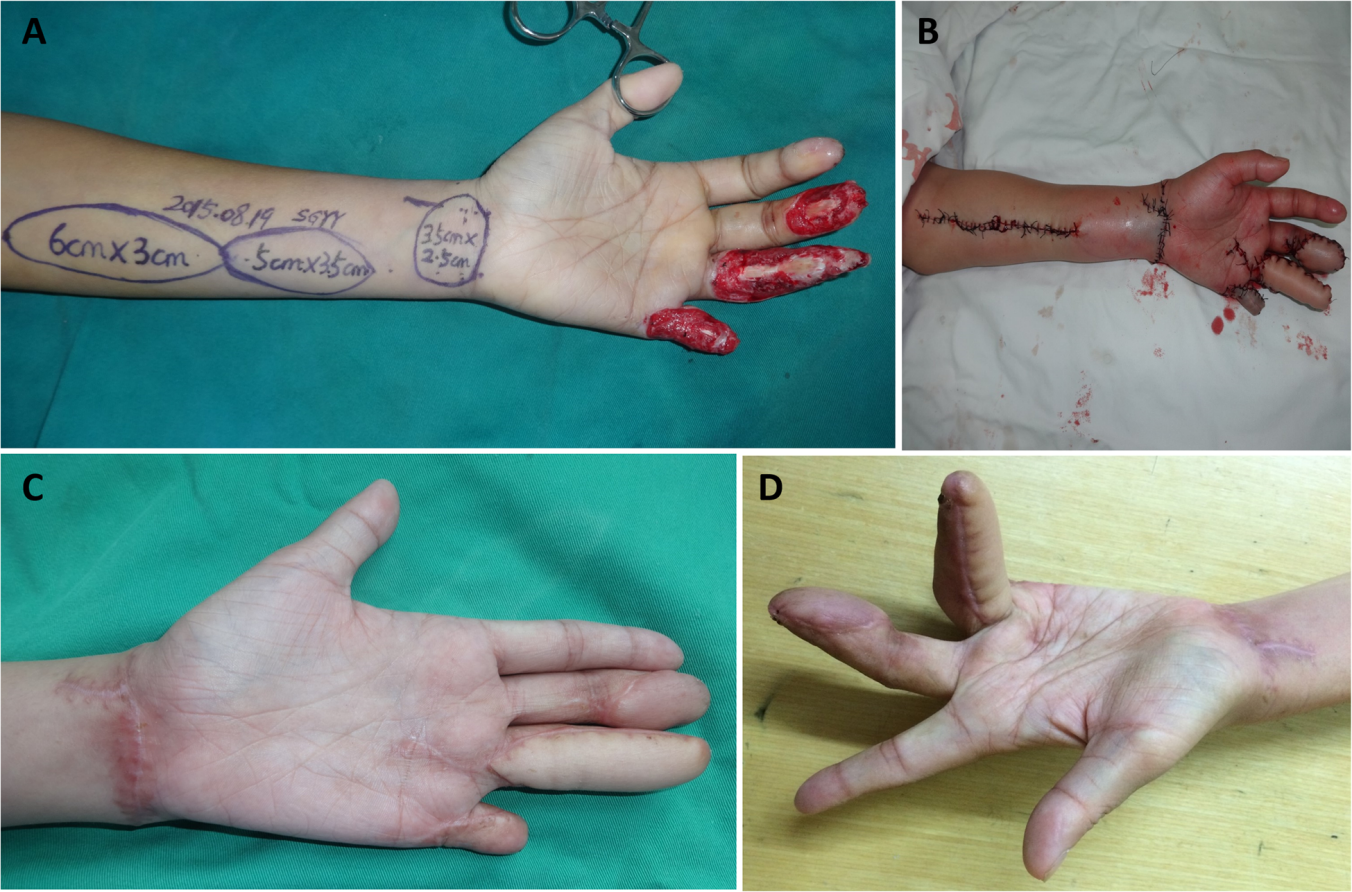
One patient had three fingers (middle finger, ring finger, and little finger) severely injured, and underwent three flap procedures (one RASPB flap and two artery perforating branch flaps from forearm). **a** Three fingers had soft tissue defects with tendons exposed. A transverse wrist crease flap combined with two artery perforating branch flaps from forearm was designed according to the wound. **b** The wound was covered with the flaps, and the donor site was closed primarily. **c**, **d** After postoperative 31 months, the appearance of injured fingers was satisfying, and the function was fair to good

**Fig. 4:**
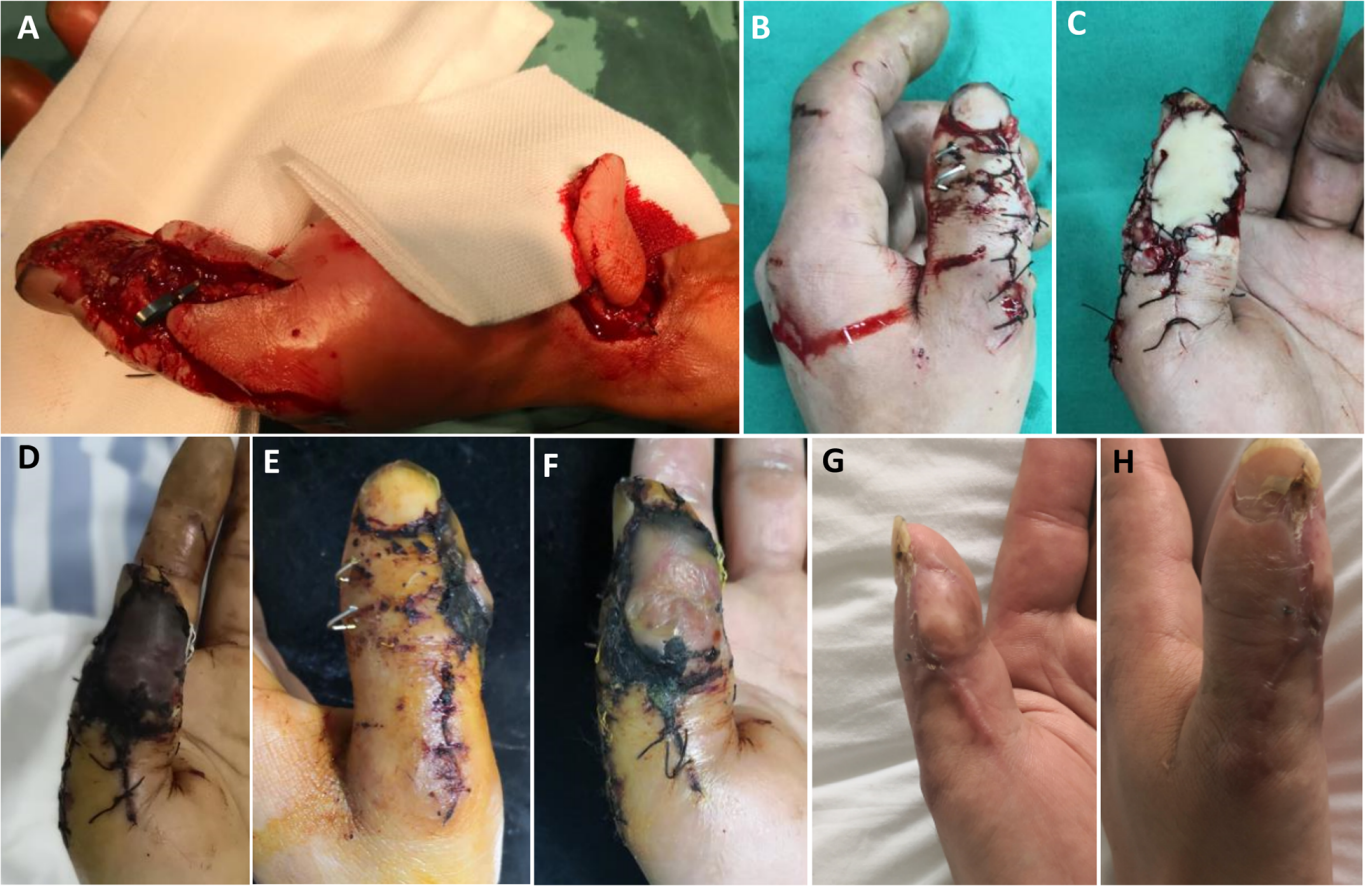
Vascular crisis appeared in one case, and the wound healed gradually after wound dressing for nearly 1 month. **a** The thumb had soft tissue defects with the tendon and bone exposed. A transverse wrist crease flap was designed and harvested according to the wound. **b**, **c** The wound was covered with the flap. **d** After postoperative 2 days, vascular crisis appeared. **e**, **f** After postoperative changing dressing for 2 weeks, the circulation of the flap was improved. **g**, **h** After postoperative 26 months, the appearance of the injured fingers was satisfying, and the function was good

All the patients were regularly followed up postoperatively for more than 24 months (range, 24–33 months). The soft tissue defects of the fingers were all covered with transverse wrist crease flap successfully, and the flaps in 71 patients survived completely without ischemia. The mean area of the donor site was 3.6 cm × 2.6 cm (length × width), and all the incisions on the donor zone could be sutured directly. One patient had three fingers (middle finger, ring finger, and little finger) severely injured and underwent three flap procedures (one RASPB flap and two artery perforating branch flaps from the forearm) (Fig. 3). Vascular crisis appeared in one case, and the wound healed gradually after changing wound dressing for nearly 1 month (Fig. 4). Slight infections of wounds appeared in eight cases, and the wound healed after changing wound dressing for 2 weeks. There were no complications in the donor site, like infection and poor wound healing. All the patients returned to their daily life and work, and all were satisfied with the function of the injured finger. At the last follow-up, the mean static two-point discrimination was 9.6 ± 2.4 mm on the injured finger and 4.3 ± 0.8 on the contralateral corresponding finger. The motion range of the distal interphalangeal joint and proximal interphalangeal joint on the injured finger were 72.5 ± 23.3% and 78.7 ± 32.5% of the contralateral corresponding finger, respectively. The outcomes of patient self-evaluations were good in 53 cases and fair in 19 cases. The postoperative following-up results of the patients are shown in Table 2.

**Table 2:** Postoperative follow-up results of the patients

Parameter	
Complications, no.	
Vascular crisis	1 (thumb)
Slight infection	8
Follow-up time, months	Range, 28.2 ± 2.4
Static two-point discrimination, millimeters	
Injured finger	Mean, 9.6 ± 2.4
Contralateral corresponding finger	Mean, 4.5 ± 0.8
Joint range of motion, % of the contralateral corresponding finger	
Proximal interphalangeal joint (or metacarpophalangeal joint of thumb)	Mean, 78.7 ± 32.5%
Distal interphalangeal joint (or interphalangeal joint of thumb)	Mean, 72.5 ± 23.3%
Grade of patient self-evaluation scores	
Good	53
Fair	19

## Discussion

It is a common problem that traumatic soft tissue defects of the finger need to be reconstructed with flap procedure [17–19]. The flap procedure should not produce severe side effects on the donor site and should be suitable to reconstruct the soft tissue defects. Traumatic soft tissue defects of the finger combined with exposure of the joints, bones, or tendons usually happen emergently and need to be covered with flap completely [20, 21]. Neurovascular island flaps, cross-finger flaps, V–Y advancement flaps, and dorsal metacarpal artery flaps have been previously reported to be applied to reconstruct the soft tissue defects of the fingers [22–24]. However, they all have responding limitations. The advanced distance of V–Y advancement flaps is limited, and this flap is usually used to cover the wound with a length less than 1.5 cm. The cross-finger flaps include a two-stage procedure, can produce morbidity on the donor finger, and are not available for multiple finger injuries [25, 26]. Therefore, V–Y advancement flaps or rotation flaps are usually the first choice, and RASPB flap can be used to treat wounds that V–Y advancement flaps or rotation flaps could not cover.

Free thenar or radial mid-palmar island flaps were reported previously, and they are also SPBRA flaps [27–31]. However, these flaps can cause obvious scarring on the thenar eminence, and it would affect the function of the hand. The transverse wrist crease flap is not harvested from the thenar eminence, and the scar on the wrist is not obvious. This flap is harvested and transferred on the same upper limb. In this procedure, the major artery was protected, and only SPBRA was sacrificed. This flap includes the median nerve palmar cutaneous branch, and it can be used as a vascularized nerve graft. When facing soft tissue defects of the fingers with digital nerve defect, we would perform end-to-end neurorrhaphy of the median palmar cutaneous branch and the digital nerves. Sensory reconstruction of the fingers is important for patients to recover feelings, which plays a key role in the patients’ daily life. The diameter of the SPBRA origin site is similar to that of the digital arteries, and they can be end-to end anastomosed expediently. Doppler ultrasonography was used to detect variations of the SPBRA preoperatively, and it can also help to find the origin of the SPBRA intraoperatively. For experienced surgeons, it usually takes approximately 30 min for harvesting RASPB flap and another 1 h for anastomosis of digital artery, superficial vein, and digital nerve. Therefore, we can usually complete all the procedure within 2 h. Previously, there are have been several studies reporting the clinical effects of this flap for reconstructing finger defects [5–7]. But these studies only include less than 15 cases and reported limited experience of just a single hospital. The safety and clinical effects are still needed to be validated with further studies including more patients and centers.

In this study, 72 consecutive patients with soft tissue defects of the fingers from six hospitals underwent the transverse wrist crease flap surgery. The flaps in 71 patients survived completely without ischemia. Only one case had vascular crisis, and the wound healed gradually after wound dressing for nearly 1 month. Slight infections of wounds appeared in eight cases. The injured fingers restored some sensory and motor functions. There were no complications in the donor site. All patients returned to their daily life and work and were satisfied with the function of the injured finger. Patient self-evaluations were good in 53 (73.6%) cases and fair in 19 (26.4%) cases. These results showed the transverse wrist crease flap technique is safe for the donor site and is effective for repairing the soft tissue defects of finger. Some surgeons may concern that the scar of this flap may be interpreted as a suicidal attempt, and patients would be unsatisfied with this procedure. We think it is really a good question. When patients signed an informed consent form for the operation, they were fully informed of the situation that the scar may be interpreted as a suicidal attempt. With preoperative detailed explanation, patients would understand and not be unsatisfied with the scar. Judging from our experience in treatment, we think the esthetic results obtained are better than non-microsurgical flaps. However, it should be verified with further clinical research. Theoretically, there may exist a neuroma of palmar cutaneous branch of the median nerve in some patients. But this is related to the quality of the neural anastomosis. In our clinical work, no patients complained pain or numbness of this zone. To make sure whether there is neuroma of palmar cutaneous branch of the median nerve, nerve ultrasound is recommended. Ischemic flaps are not rarely faced by microsurgeons. Before the flap pedicle was cut in this study, we could check if there was a twist in the pedicle of the flap. After end-to-end anastomosis of the vessel was performed, we could keep the flap warm and use vasoactive medicine. If those measures do not work, a second operation will be performed to find out if there is thrombosis in the vessels.

The free neurovascular transverse wrist crease flap technique also has its limitations. The arterial pedicle of this flap has a relatively short length. There is no accurate data on inclusion criteria or on exclusion criteria as regards lesion size yet. Surgeons judge it according to clinical experience. The flap can cover small- to medium-sized defects. The largest area reported previously is 5.1 cm × 3.4 cm, and the donor site needs skin grafting [10]. The SPBRA sometimes has variation and may result in inadequate length, and this makes the flap surgery not suitable. Under this condition, intraoperative treatment strategy should be changed accordingly.

## Conclusions

In conclusion, the clinical outcomes of the free neurovascular transverse wrist crease flap for repairing soft tissue defects of the fingers in multiple centers indicate the free neurovascular transverse wrist crease flap is a safe and effective technique for reconstructing soft tissue defects of the fingers. When there is more than one finger having soft tissue defects, the transverse wrist crease flap can also be performed combined with other flap procedures.

## Data Availability

The datasets used and analyzed during the current study are available from the corresponding author on reasonable request.

## Abbreviations

RASPB: Radial artery superficial palmar branch
ROM: Range of motion
